# The Burden and Impact of Vertigo: Findings from the REVERT Patient Registry

**DOI:** 10.3389/fneur.2013.00136

**Published:** 2013-10-02

**Authors:** Heike Benecke, Sam Agus, Daniel Kuessner, Gordon Goodall, Michael Strupp

**Affiliations:** ^1^Abbott Laboratories GmbH, Hannover, Germany; ^2^Abbott Products Operations AG, Allschwil, Switzerland; ^3^Department of Neurology, German Centre for Vertigo and Dizziness (IFB LMU), University Hospital Munich, Munich, Germany

**Keywords:** vertigo, economic burden, Meniere’s disease, registry, healthcare resource

## Abstract

**Objective:** Despite the high prevalence of vertigo globally and an acknowledged, but under-reported, effect on an individual’s wellbeing, few studies have evaluated the burden on healthcare systems and society. This study was aimed to quantitatively determine the impact of vertigo on healthcare resource use and work productivity.

**Methods:** The economic burden of vertigo was assessed through a multi-country, non-interventional, observational registry of vertigo patients: the Registry to Evaluate the Burden of Disease in Vertigo. Patients included were those with a new diagnosis of Meniere’s disease, benign paroxysmal positional vertigo, other vertigo of peripheral vestibular origin, or peripheral vestibular vertigo of unknown origin.

**Results:** A total of 4,294 patients at 618 centers in 13 countries were included during the registry. Of the 4,105 patients analyzed, only half were in employment. Among this working patient population, 69.8% had reduced their workload, 63.3% had lost working days, and 4.6% had changed and 5.7% had quit their jobs, due to vertigo symptoms. Use of healthcare services among patients was high. In the 3 months preceding Visit 1, patients used emergency services 0.4 ± 0.9 times, primary care consultations 1.6 ± 1.8 times, and specialist consultations 1.4 ± 2.0 times (all mean ± SD). A mean of 2.0 ± 5.4 days/patient was also spent in hospital due to vertigo.

**Conclusion:** In addition to the negative impact on the patient from a humanistic perspective, vertigo has considerable impact on work productivity and healthcare resource use.

## Introduction

Vertigo is defined as a perceived movement of either one’s own body, such as swaying or rotation, or of the environment, or both, in the absence of physical movement (1–3) and secondary symptoms include cold sweating, nausea, and vomiting (4). Vertigo and dizziness are most often caused by different vestibular disorders, such as benign paroxysmal positional vertigo (BPPV), Meniere’s disease (MD), and other vertigo of peripheral vestibular origin (2, 5). Causes of vertigo are multi-faceted and can be due to a dysfunction of the peripheral or central vestibular system (2, 3), but may also be psychogenic.

Vertigo is one of the most common conditions with which patients present to physicians and its incidence increases with age (3, 6, 7). Despite a lifetime prevalence of dizziness and vertigo estimated at 20–30% and 1-year prevalence estimate for vertigo of 4.9% (8), the healthcare burden of vertigo is still relatively under-reported due to the unpredictability of attacks and the nature of the disease (4).

Although often perceived as a mild physical disorder with low morbidity, the psychological impact of vertigo can lead to a substantial impact on the individual’s lifestyle and behavior (4). For example, research suggests that patients have had to quit, change, or modify their jobs due to their symptoms (4) suggesting a considerable impact on quality of life (6, 9).

Despite this, few studies have attempted to quantify the economic burden of vertigo including the impact on work productivity and healthcare resource use (5, 9). Of the studies that have been published, some were constrained by the context of a clinical trial (10), and others were small scale local-level studies where information was collected retrospectively (11). Therefore, the objective of the Registry to Evaluate the Burden of Disease in Vertigo (REVERT) was to help estimate the burden associated with vertigo from a multi-country perspective. In a previous publication, we reported the clinical and demographic features of the patient population included in REVERT, before treatment and after 6 months of treatment (12). Here we focus specifically on the economic impact on healthcare resource use and the cost implications in terms of lost work productivity.

## Materials and Methods

### Study design and population

Data were collected through a multi-country non-interventional registry of vertigo patients. Invitations to participate in the study were sent to centers in 112 countries. Of these, 618 centers in 13 countries agreed to participate (Algeria, Czech Republic, Egypt, Germany, Hungary, Lithuania, Malaysia, Morocco, Russia, Slovenia, South Africa, Tunisia, and Ukraine). Ethical approval for the study was gained in each country where necessary, in accordance with the ethical standards laid down in the 1964 Declaration of Helsinki.

Participating healthcare professionals, including Ear Nose and Throat Specialists (ENTs), Neurologists, General Practitioners, and Accident and Emergency Physicians, documented the characteristics and treatment of the next two consecutive patients who presented with a diagnosis of vertigo of peripheral origin (either as first diagnosis for the enrolling physician or as a new consultation). This included patients with other vertigo of peripheral vestibular origin, BPPV, peripheral vestibular vertigo of unknown pathology, or MD. No further inclusion criteria were defined and there were no specific instructions regarding treatment implementation, diagnostics, or on-going examination schedule. This was to allow for non-biased real-world data collection (rather than “research procedures”). Patient consent was obtained prior to inclusion.

Patients were examined at baseline (Visit 1) and at a scheduled 6-month follow-up visit (Visit 2). This report is based on the analysis of data at Visit 1, focusing on the healthcare resource use and working status by diagnosis and severity in the 3 months prior to Visit 1.

Paper-based standardized case report forms (CRFs) were translated into the local language where applicable. The questions covered demographic information, medical history, treatments prior to inclusion in the registry, prescription of a treatment after enrollment (prescription after enrollment was at the discretion of the participating physician and was not mandatory), and Clinical Global Impression score (CGI). The CGI is a three-item observer-rated scale and is used in this registry to measure illness severity (CGI-S) (13). CGI-S was assessed at baseline based on the patient’s description of their condition for the 2 days preceding the visit and classed as, “normal, not ill at all,” “borderline ill,” “mildly ill,” “moderately ill,” “markedly ill,” “severely ill,” “among the most extremely ill patients.”

Patients reported the frequency and duration of specific healthcare service use [hospitalization, emergency service use, and general practitioner (GP) or specialist consultation] due to vertigo symptoms during the 3 months preceding Visit 1. Patients also reported their working status and work days lost in the past 3 months due to vertigo. Healthcare utilization data collected in this registry is therefore not a long-term indication of usage. Work productivity data collected in this registry is not a long-term indictor of productivity loss. Furthermore, data were collected to help understand the real-world complexities of diagnosis, prognosis, and treatment of vertigo.

### Statistical analysis

Statistical analysis is consistent with the limitations of research in a naturalistic setting. All summary statistics were performed using STATA software (special edition 10.0). All analysis variables were assessed descriptively; discrete data were summarized presenting counts and percentages. Statistical significance was not determined in this study. Missing values were not included when calculating percentages; however, answers of “not applicable” were included. Only patients who attended Visit 1 were included in the analysis. Continuous data were summarized by the following summary statistics: number of subjects with non-missing values, number of subjects with missing values, mean, and standard deviation.

## Results

### Demographics

The registry ran from 20th April 2007 to 15th August 2009. A total of 4,294 patients entered the registry, of which 4,105 reported both diagnosis and economic data at Visit 1 and were included in this analysis. A greater proportion of females (65.4%) than males were included in the registry. Table 1 provides an overview of the study population at Visit 1. The most common diagnosis was “other vertigo of peripheral vestibular origin” (37.2%), followed by BPPV (26.9%), peripheral vestibular vertigo of unknown origin (20.5%), and MD (15.4%).

**Table 1:** **Study population in REVERT registry at Visit 1 (*n* = 4,105)**.

Variable	*n* (%)
**COUNTRY (*n* = 4,105)**	
Algeria	274 (6.7)
Czech Republic	559 (13.6)
Egypt	168 (4.1)
Germany	99 (2.4)
Hungary	1,320 (32.2)
Lithuania	202 (4.9)
Malaysia	354 (8.6)
Morocco	118 (2.9)
Russia	253 (6.2)
Slovenia	130 (3.2)
South Africa	34 (0.8)
Tunisia	185 (4.5)
Ukraine	409 (10.0)
**GENDER (*n* = 4,079)^a^**	
Male	1,417 (34.7)
Female	2,662 (65.3)
**AGE RANGES, YEARS (*n* = 4,093)^b^**	
≤20	29 (0.7)
21–30	181 (4.4)
31–40	437 (10.7)
41–50	799 (19.5)
51–60	1,079 (26.4)
61–70	816 (19.9)
71–80	548 (13.4)
>80	204 (5.0)
**DIAGNOSIS (*n* = 4,048)^c^**	
Meniere’s disease	625 (15.4)
Benign paroxysmal positional vertigo	1,090 (26.9)
Other vertigo of peripheral vestibular origin	1,504 (37.2)
Peripheral vestibular vertigo of unknown origin	829 (20.5)
**SIGNIFICANT MEDICAL HISTORY (*n* = 3,676)^d^**	
Cardiac/vascular disease	1,702 (46.3)
Hormonal dysfunction	634 (17.2)
Neurologic disorder	338 (9.2)
Cranial trauma	204 (5.5)
Neoplasm	133 (3.6)
Psychiatric disorder	558 (15.2)
Drug/alcohol abuse	107 (2.9)

^a^*n* = 26 missing data on gender;

^b^*n* = 12 missing data on age;

^c^*n* = 57 missing data on diagnosis;

^d^*n* = 429 missing data on medical history.

### Impression of severity

At Visit 1, 43% patients were classified by their clinicians as “moderately ill,” with 22.2% “mildly ill,” 20.0% “markedly ill,” 7.4% “borderline ill,” and 4.1% “severely ill.” This classification of symptoms was equal across all age groups and for both genders (data not shown).

### Medical therapy before enrollment

Reported medicinal products used prior to study enrollment were betahistine (26.6%), piracetam (11.5%), and gingko biloba (5.7%). Other therapies included benzodiazepines (4.0%), calcium antagonists (2.2%), neuroleptics (1.9%), antihistamines (1.7%), and homeopathic medication (1.1%). Co-prescription of treatments was common. Prescription of a treatment after enrollment to the registry was at the discretion of the participating physician and was not mandatory.

### Healthcare utilization

**Figure 1:**
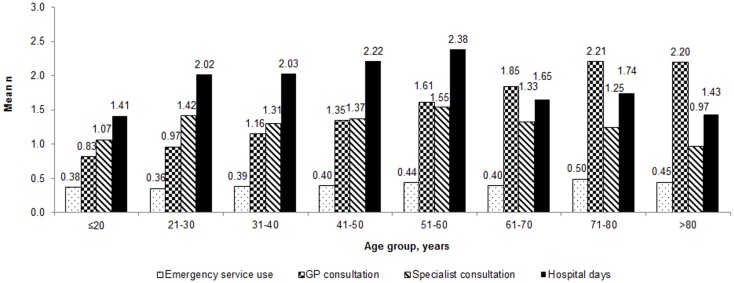
**Healthcare utilization by patients with vertigo during the 3 months before Visit 1 by age group**.

Overall patient-reported mean healthcare use due to vertigo over the previous 3 months was 0.4 (SD ± 0.9) utilizations of emergency services, 1.6 ± 1.8 GP consultations, 1.4 ± 2.0 specialist consultations (e.g., ear-nose-throat specialist or neurologist), and a mean of 2.0 ± 5.4 days spent in hospital due to vertigo. Figure 1 shows the variation in healthcare resource use by age.

The number of GP consultations during the 3 months prior to Visit 1 was 0.8 in those ≤20 years of age and was 2.2 in those aged >80 years. Those patients between the ages of 51 and 60 years spent on average 2.4 days in hospital compared to 1.4 in those aged ≤20.

Healthcare resource use was also recorded in relation to diagnosis. The mean number of hospital days due to vertigo (during the 3 months before Visit 1) was 2.7 for those diagnosed with other vertigo of peripheral vestibular origin, compared to 1.4 for those diagnosed with peripheral vestibular vertigo of unknown origin (Table 2). The mean number of GP consultations, during the 3 months prior to Visit 1, was 1.8 for those diagnosed with peripheral vestibular vertigo of unknown origin, whilst those with MD and BPPV had on average 1.4 consultations.

**Table 2:** **Healthcare utilization during the 3 months before Visit 1 by diagnosis**.

Diagnosis	Emergency service use	GP consultations	Specialist consultations	Days spent in hospital
	mean (±SD)			
Meniere’s disease	0.53 (0.99)	1.36 (1.83)	1.46 (1.45)	1.63 (4.80)
Benign paroxysmal positional vertigo	0.40 (0.85)	1.42 (1.75)	1.37 (3.06)	1.73 (5.73)
Other vertigo of peripheral vestibular origin	0.41 (0.82)	1.78 (1.88)	1.46 (1.62)	2.65 (5.82)
Peripheral vestibular vertigo of unknown origin	0.40 (0.81)	1.83 (1.72)	1.14 (1.21)	1.37 (3.89)

MD, Meniere’s disease; BPPV, benign paroxysmal positional vertigo; GP, general practitioner; SD, standard deviation.

**Figure 2:**
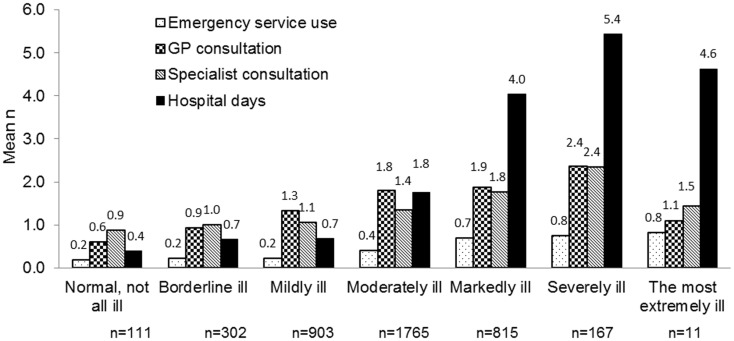
**Healthcare utilization by patients with vertigo during the 3 months before Visit 1 by illness severity**.

The severity of disease was also captured in relation to healthcare resource use in the 3-month period prior to Visit 1 (Figure 2). Those classed as “severely ill” spent on average 5.4 days in hospital compared with 0.4 days for patients assessed as “normal, not at all ill.” Similarly the mean number of consultations with GPs or Specialists was 0.6 and 0.9 respectively for those assessed as “normal, not at all ill,” compared to 2.4 consultations (for both GP and specialists) for those who were assessed as “severely ill” (Figure 2). Owing to the low number of patients who were “the most extremely ill” no detailed conclusions can be drawn from this group.

**Figure 3:**
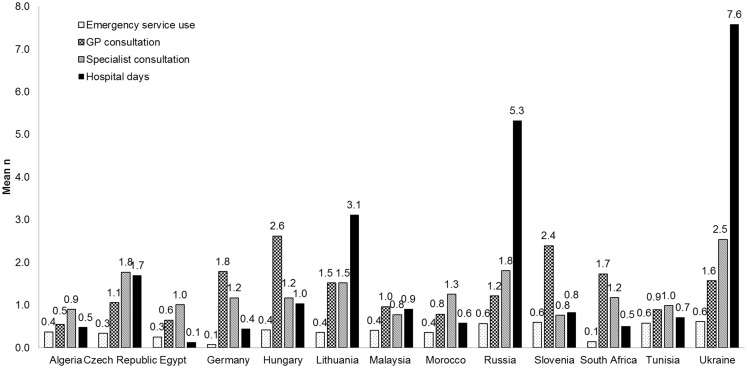
**Healthcare utilization by patients with vertigo during the 3 months before Visit 1 by country**.

The healthcare utilization due to vertigo during the 3 months before Visit 1 was also captured in relation to the different countries studied (Figure 3). Patients in the Ukraine and Russia spent on average 7.6 and 5.3 days respectively in hospital due to vertigo compared with an average of 0.1 days in Egypt. The numbers of GP consultations ranged from means of 2.6 in Hungary, to 0.5 in Algeria. The use of emergency services ranged from a mean of 0.6 occasions for Tunisia and the Ukraine to 0.1 occasions in Germany.

### Change of work status and lost days at work

At Visit 1, about half of all the subjects were in work, either employed (39.6%) or self-employed (9.1%). Over one third were retired (36.1%) and 10.8% were unemployed. An undefined status of “other” was documented for 4.5% of subjects as their current occupation. Differences were found across countries in terms of working status. These data indicate the working status during the 3-month period prior to Visit 1 and are not a long-term indication of status.

A total of 69.8% patients reported they had reduced their workload and 63.3% lost working days due to their vertigo, whilst 4.6% reported changing their job and 5.7% quit work completely as a consequence of their condition. The impact of age on working status is shown in Table 3.

**Table 3:** **Impact of vertigo on the working status of the employed population, by age group, diagnosis, and illness severity in the 3 months before Visit 1 (% of employed patients)**.

	Patients, *n*	Reduced workload	Loss of working days	Change of job	Quit job
		% of employed patients			
**AGE RANGES, YEARS**					
≤20	10	70.0	60.0	0.0	0.0
21–30	100	64.7	63.6	5.3	7.4
31–40	292	72.4	64.1	5.9	6.3
41–50	533	68.0	64.9	5.4	5.4
51–60	567	70.8	61.6	3.5	5.9
61–70	102	72.7	63.7	3.0	4.0
≥70	11	60.0	45.5	0.0	9.1
**DIAGNOSIS**					
MD	269	72.0	69.7	9.2	8.9
BPPV	462	68.1	60.2	4.0	6.5
Other vertigo of peripheral vestibular origin	593	72.8	65.3	3.3	4.7
Peripheral vestibular vertigo of unknown origin	288	64.2	56.6	2.8	3.2
**GLOBAL IMPRESSION OF SEVERITY**					
Normal, not all ill	45	38.6	31.1	0.0	0.0
Borderline ill	138	46.3	37.2	1.5	1.5
Mildly ill	410	51.7	41.8	0.7	1.7
Moderately ill	612	76.7	70.0	4.7	5.5
Markedly ill	342	90.7	87.6	9.2	11.2
Severely ill	53	88.7	92.5	18.0	22.0
The most extremely ill	5	80.0	40.0	0.0	20.0

MD, Meniere’s disease; BPPV, benign paroxysmal positional vertigo.

Disease severity was also recorded in relation to working status in the employed population (Table 3). Of those patients assessed as “severely ill,” 88.7% reduced their workload, 92.5% lost working days, 18% changed their job, and 22.0% quit work completely (Table 3). Of those assessed as “normal, not at all ill,” 38.6% reduced their workload and 31.1% lost working days. No patients changed or quit their job. The low number of employed patients who were classed as “the most extremely ill” preclude any detailed conclusions being drawn from this group. Responses to questions on working status were not mutually exclusive and multiple answers were allowed. Finally, Table 4 shows the number of lost work days in the past 3 months before Visit 1 by country.

**Table 4:** **Mean number of lost work days among employed patients in the past 3 months before Visit 1 by country**.

Country	Patients, *n*	Lost work days in the past 3 months before Visit 1	
		Mean (SD)	Min, Max
Algeria	68	11.2 (9.3)	1, 60
Czech Republic	132	13.1 (14.6)	1, 90
Egypt	48	9.5 (13.5)	1, 90
Germany	22	26.7 (39.5)	1, 90
Hungary	161	13.2 (10.9)	2, 75
Lithuania	73	15.7 (17.0)	1, 90
Malaysia	79	8.7 (16.1)	1, 90
Morocco	28	13.9 (16.6)	2, 90
Russia	131	13.7 (10.6)	1, 66
Slovenia	36	15.8 (15.5)	1, 84
South Africa	16	9.7 (14.5)	1, 60
Tunisia	56	9.5 (12.1)	1, 90
Ukraine	132	15.8 (15.4)	1, 90

SD, standard deviation.

## Discussion

Naturalistic data on the burden of vertigo are limited, therefore the aim of the REVERT registry was to prospectively examine, in a large patient population, the impact of vertigo on work performance and healthcare utilization.

The results of this registry suggest that in the 13 countries studied, high frequencies of GP consultations, specialist consultations, and emergency services usage are likely to impose a considerable burden on healthcare providers. Furthermore, these data suggest that healthcare use may be influenced by increasing age and disease severity; therefore, treatments that can alter the natural course of the disease may lead to reductions in the burden of disease even if they do not provide a cure.

Due to the unique nature of our study and its observational design, we were not able to perform comparisons between patients with vertigo on one side and healthy patients on the other side. Also, to the best of our knowledge, there are no similar studies in the literature which would allow a comparison of our findings with previously published data. However, the overall findings of this study support those of other local-level studies. For example, a recent study on the epidemiology of vertigo in Taiwan found that vertigo is a major health burden among the general adult population and tends to recur, particularly among older women (14). In the United States, dizziness is estimated to account for 5.0% of walk-in clinic visits and there are 2.6 million visits to emergency departments due to dizziness annually, which accounted for 3.3% of all emergency department visits during 1993–2005 (15). In addition, Neuhauser et al. evaluated the burden of vertigo in Germany in a study of 1,003 patients (9). The results from this study are in line with these findings and demonstrate that patients suffering from vertigo frequently consulted medical practitioners, often required sick leave and had interruptions to their daily activities (9). While this study indicated that both dizziness and vertigo are frequent symptoms in general practice and inflict a considerable health care burden, it is also noted that vertigo disorders are associated with lower quality of life compared to control subjects. The authors also indicated that the underestimation of dizziness and vertigo symptoms and resultant effects, are reflected in the fact that large percentages of the underlying disorders remain under-diagnosed and, therefore, are presumed to be insufficiently treated.

From the perspective of those individuals who were in employment, data from the REVERT registry highlight the impact of vertigo on work performance. For example, 70.0% of the working population had reduced their workload and 63.0% had lost working days in the 3 months before the baseline visit. On average, as many as 14 working days were lost due to vertigo during the prior 3 months among those in employment. The results suggest that work performance and employment status are also affected by the specific diagnosis and severity of vertigo symptoms in patients who are employed, although differences exist presumably due, at least in part, to variances in the social care systems of the respective countries. These results are consistent with the limited published data, with Skøien et al. reporting that vertigo symptoms can lead to long-term work absence and an increased risk of requiring a disability pension later in life (5).

When there is limited evidence on the healthcare- and work-burden of disease, it is standard practice to compare a condition to a disease with a similar progression. Aspects of vertigo are mirrored in other conditions where low mortality but high morbidity translates into a negative impact on daily activities. For example, people with chronic lower back pain experience persistent discomfort for which temporary pain relief is often ineffective and insufficient. This leads to a decrease in productivity due to absenteeism or presenteeism which translates into high indirect costs (i.e., to society and employers) (15, 16). Furthermore, in the USA, back pain is one of the most frequent reasons for visits to physicians and hospitalizations, leading to a high burden on the healthcare system (16).

Registry-based, observational studies have several well-documented limitations. The drawbacks of uncontrolled real-world evidence include insufficient quality and depth to data, potential sources of bias, and significant missing data. For example, in this study healthcare resource use and working status are based on patient memory which is subject to recall bias. These answers were not cross-checked with medical and social records, and the diagnoses were not universally validated, which could mean that these differed between both institutions and countries. There is also no clarity on diagnosis definitions, which could mean that these differed by center and country: in order to try to at least partly overcome this limitation, we created the diagnostic category “Other vertigo of peripheral vestibular origin.” This category might lack specificity, but has the advantage of avoiding an inconsistent attribution of patients to various, not well defined diagnostic categories.

Participating countries and centers were selected based on willingness to contribute and therefore do not provide global coverage and do not necessarily provide a representative sample of each country, which is a general limitation of research in a naturalistic registry setting. For example, Hungary recruited far more patients than any other country; such disparities in recruitment confer the risk of a non-representative patient population. These disparities might also have led to some inconsistencies in the results: for example, we observed major differences in the amount of lost working days across countries. Furthermore, this was a multinational study involving countries with different cultures and approaches to health and social care, and this must be considered as potentially contributing to the heterogeneity in the data. Despite these drawbacks, registries studies, including this one, generate data of vast potential and are important for collecting, classifying, and linking disease-specific information. While clinical trials give generate data in controlled conditions, registries can provide real-world data on the natural history of disease, in a more diverse patient population over a long-period (16).

In conclusion, the results from the REVERT registry provide some valuable insight into the potential personal, societal, and economic burden of vertigo. This may be of particular relevance in countries where a societal view of healthcare is applied since an improvement in the management of vertigo patients may have effects not only on the healthcare system but on society overall.

## Conflict of Interest Statement

Heike Benecke is an employe of Abbott Products GmbH, Germany. Sam Agus, Gordon Goodall, and Daniel Kuessner are former employes of Abbott Products Operations AG, Switzerland. Michael Strupp is employed by the University Hospital Munich (Department of Neurology and IFB LMU). Michael Strupp has received honoraria from Abbott Laboratories, UCB, Biogen Idec, GlaxoSmithKline, Hennig, and Ratiopharm. The authors do not report any conflict of interest with regards to the contents of this study other than those stated.
